# Mutation Induced Extinction in Finite Populations: Lethal Mutagenesis and Lethal Isolation

**DOI:** 10.1371/journal.pcbi.1002609

**Published:** 2012-08-02

**Authors:** C. Scott Wylie, Eugene I. Shakhnovich

**Affiliations:** Department of Chemistry and Chemical Biology, Harvard University, Cambridge, Massachusetts, United States of America; Pennsylvania State University, United States of America

## Abstract

Reproduction is inherently risky, in part because genomic replication can introduce new mutations that are usually deleterious toward fitness. This risk is especially severe for organisms whose genomes replicate “semi-conservatively,” e.g. viruses and bacteria, where no master copy of the genome is preserved. Lethal mutagenesis refers to extinction of populations due to an unbearably high mutation rate (*U*), and is important both theoretically and clinically, where drugs can extinguish pathogens by increasing their mutation rate. Previous theoretical models of lethal mutagenesis assume infinite population size (*N*). However, in addition to high *U*, small *N* can accelerate extinction by strengthening genetic drift and relaxing selection. Here, we examine how the time until extinction depends jointly on *N* and *U*. We first analytically compute the mean time until extinction (*τ*) in a simplistic model where all mutations are either lethal or neutral. The solution motivates the definition of two distinct regimes: a survival phase and an extinction phase, which differ dramatically in both how *τ* scales with *N* and in the coefficient of variation in time until extinction. Next, we perform stochastic population-genetics simulations on a realistic fitness landscape that both (i) features an epistatic distribution of fitness effects that agrees with experimental data on viruses and (ii) is based on the biophysics of protein folding. More specifically, we assume that mutations inflict fitness penalties proportional to the extent that they unfold proteins. We find that decreasing *N* can cause phase transition-like behavior from survival to extinction, which motivates the concept of “lethal isolation.” Furthermore, we find that lethal mutagenesis and lethal isolation interact synergistically, which may have clinical implications for treating infections. Broadly, we conclude that stably folded proteins are only possible in ecological settings that support sufficiently large populations.

## Author Summary

Most spontaneous mutations hurt organismal fitness, e.g. by destabilizing proteins. In many species, the normal mutation rate is strikingly high: on the order of one per genome per replication. In the face of these mutations, how can proteins maintain their native structure, and how can populations of organisms avoid extinction? Are there physics-based limits on how large the mutation rate of any species can be before the onslaught of mutations outpaces natural selection and melts-down proteins? Here, we address these questions with a computational model that combines protein folding thermodynamics with individual-based population genetics simulations. We calculate a theoretical “speed limit” equal to a few mutations per genome per replication—near the mutation rate of RNA viruses. Additionally, we find that the speed limit can be much lower in small populations where “random genetic drift” is strong. Thus, we conclude that stably folded proteins are only possible in ecological settings that support sufficiently large populations. These findings may have clinical implications for treating viral infections with drugs that elevate the viral mutation rate.

## Introduction

On average, mutations hurt organismal fitness, e.g. by destabilizing proteins. Thus, left unchecked, new mutations tend to erode fitness and endanger the long-term survival of any species. Fortunately, natural selection usually balances against mutational genetic decay by rewarding the fit and weeding out the unfit. However, when the genomic mutation rate (i.e. the expected number of mutations per genome duplication) exceeds a critical value (*U_crit_*), mutation outpaces selection, causing population extinction in a process known as “lethal mutagenesis” [1]. Lethal mutagenesis is important both theoretically [2]–[7] and clinically, where drugs (e.g. Ribavirin) can extinguish pathogens, especially RNA viruses, by elevating the mutation rate beyond *U_crit_* [1], [8]–[14].

Aside from mutation rate, population size (*N*) also plays an important role in extinction. All existing estimates of *U_crit_* assume that *N = ∞* [2]–[4], so that extinction can be modeled with relatively simple deterministic equations. In contrast, every real population has only finitely many members and is consequently subjected to “random genetic drift,” i.e. stochastic fluctuations in birth-death events. More precisely, every real population of size *N* is guaranteed to experience fluctuations of order *∼1/N* reminiscent of “shot noise,” since births and deaths occur as discrete events. Upon first thought, it may seem that genetic drift merely represents a small correction to the deterministic dynamics. However, the actual behavior is dramatically more interesting: Since drift continually obfuscates fitness differences among individuals, it weakens selection and implicitly tilts the mutation-selection balance in favor of mutation [15], [16]. By this mechanism, known as “Muller's ratchet,” [17] *unbiased* birth-death fluctuations end up downwardly *biasing* mean fitness within a population. Muller's ratchet has long been studied theoretically [18]–[24] and routinely exploited experimentally to prepare low fitness lines of organisms [25]. However, the extent to which high mutation rates exacerbate Muller's ratchet en route to extinction is neither qualitatively nor quantitatively well understood. We revisit this issue and review the literature on Muller's ratchet in Discussion.

In principle, mutations can cause extinction by two distinct, though non mutually exclusive mechanisms. First, deleterious mutations might decrease the absolute birth rate of a population to such a great extent that individuals are killed by natural forces (e.g. old age, environmental stresses, etc) faster than they reproduce. Most previous studies, e.g. refs. [2], [19], [20], [22], [23], have analyzed this first scenario, which represents a struggle between a population and its environment. A second, qualitatively distinct scenario is possible for organisms that reproduce “semi-conservatively,” including all viruses and unicellular species (see Results for elaboration): Every birth event risks ruining the “original” genome with new lethal mutations, thereby reducing the census size and risking extinction [4], [26]. These dynamics represent the struggle of a population against itself. In this paper, we focus primarily on this second mode of extinction.

A sticking point for all lethal mutagenesis models is the relationship between genotype and fitness, i.e. the fitness landscape (FL). The distribution of fitness effects (DFE) among new single mutations furnishes the first order description of the FL. The second order description specifies the form of epistasis, i.e. how pairs of mutations interact to impact fitness. With few exceptions [3], [5], [7], previous studies generally assume that the DFE conforms to a simple mathematical function and make drastic simplifying assumptions regarding epistasis. In particular, most previous studies assume either no epistasis, or that mutations interact either all synergistically or all antagonistically, leaving little room for phenomena such as compensatory mutations. The motivation behind those assumptions is in part due to the large number of (unknown) parameters necessary to even write down a reasonably complex, epistatic FL.

Here, we circumvent that impasse by utilizing a previously developed approach [27] that is virtually parameter-free. We do not explicitly *impose* a DFE or a model of epistasis. Instead, those features emerge as the *output* from a biophysics-based protein folding requirement: We assume that mutations inflict fitness penalties proportional to the extent that they unfold proteins. Remarkably, this minimal assumption roughly accounts for the DFE observed in site-specific mutagenesis experiments on several viral species [27].

Here, we combine our biophysics-based FL with individual-based population-genetics simulations where extinction can only result from lethal mutations. We measured the time until extinction (*τ)* as a function of population size (*N*) and mutation rate (*U*). In accord with previous studies, we observe an ultimate mutation rate (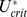
≈2.5 mutations per genome) beyond which even infinitely large populations go extinct “almost immediately” (*τ∼log(N)* generations). However, when 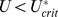, we find that *τ* depends dramatically on *N*: Small populations go extinct in *τ∼log(N)* generations, whereas large populations survive “almost indefinitely” (*τ∼e^N^/N*). The boundary between “large” and “small” populations depends on *U* and is reminiscent of a “phase transition” between survival and extinction. In addition to *τ*, the coefficient of variation (i.e. standard deviation divided by mean) also undergoes a transition from values near zero in the extinction phase to values near one in the survival phase. These results contradict the simplistic intuition that “small populations are more stochastic than large populations.”

For comparison, we also analytically solve for *τ* in a very simple model in which all mutations are either lethal or neutral. Solutions to this model clarify the meaning of extinction in finite populations and motivate our definition of survival vs. extinction phases.

## Results

### Semi-conservative birth-death-mutation model

#### Birth

This paper concerns asexual populations of replicating entities, henceforth called “cells.” “Births” occur when a mother cell gives rise to exactly two daughters and the mother simultaneously dies. In continuous time, individual cells are chosen to give birth with probability proportional to their fitness (*W*), i.e. their birth rate. See Methods for further details.

#### Death

**Figure 1:**
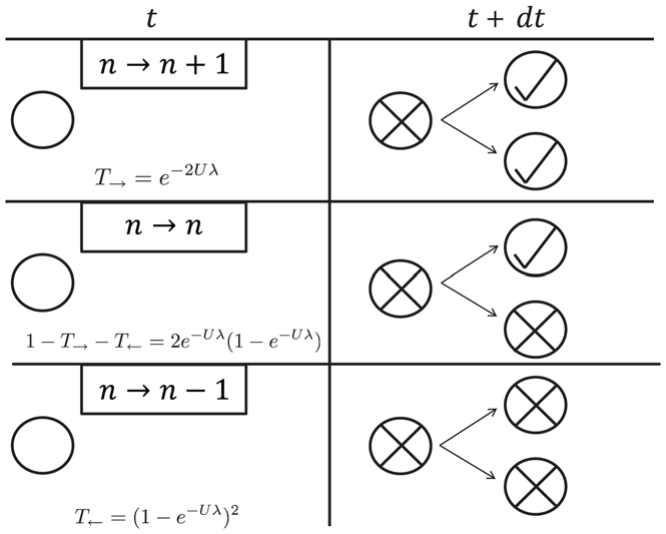
Mechanism by which the number of cells (*n(t)*) increases or decreases in the absence of natural death (*δ = 0)*. The situation before and after a birth event is shown in the left and right columns, respectively. A parent cell immediately dies after giving rise to exactly two daughters. Replication is semi-conservative: Each daughter independently acquires a Poisson distributed number of lethal mutations, with mean *Uλ*. If both daughters are free of lethal mutations (check marks), then *n(t)* increases by one (top row). If both daughters acquire lethal mutations and die (“x” marks), then *n(t)* decreases by one (bottom row). The probability of each transition is also shown in the left column. In addition to these mechanisms, cells may experience natural death with a fixed rate (*δ*).

Besides the death of mothers upon birth, death also occurs by three additional mechanisms. First, if a birth event “tries to” increase the number of cells (*n(t))* beyond a maximum number (*N)*, then a random cell is removed from the population (similar to Moran's model [28]). Note that, strictly speaking, *N* is not the population's size but rather its capacity; density-dependent mortality kicks in abruptly when *n = N*. Secondly, as described below, some cells inherit new mutations, and if any of these mutations are lethal, then that daughter cell is killed immediately. Thirdly, cells can die (with rate *δ*) of “natural” causes, e.g. old age, washout, clearance, etc, independent of replication events or lethal mutations. Extinction in real populations is likely caused by a combination of these second and third mechanisms of death. For the bulk of this paper, we focus our attention on the second source, which represents an ultimate limit to population survival. A crucial feature of this limiting regime is that extinction cannot result from low fitness per se, which merely increases the generation time. Rather, lethal mutations are the only mechanism that can cause extinction in this regime (fig. 1).

#### Mutation

Each of the two daughter cells independently can acquire (nonsynonymous) mutations during their birth, i.e. replication is semi-conservative; see below for elaboration on this crucial assumption. In particular, if both daughters inherit lethal mutations, then the census size decreases by one. Note that if a master copy of the replicating genome was preserved (i.e. replication was conservative) and the natural death rate is zero, the population could not decrease in size and extinction would be impossible. We assume that the number of new nonsynonymous mutations per cell per birth event is Poisson distributed with mean *U*, i.e. *U* is the total genomic nonsynonymous mutation rate. In general, mutations can either be lethal or else merely perturb fitness (*W*), i.e. alter the doubling time. Our hypothesis that the onset of extinction is marked by excessive lethal mutations has experimental support [29] in viruses (see Discussion).

#### Biological interpretations

Our use of the term “semi-conservative” is based on the mechanism of DNA replication during cellular binary fission: Each daughter cell inherits one of the parent's two DNA strands, which then acts as a template for (potentially erroneous) synthesis of the remaining strand. Semi-conservative replication applies to all unicellular species. Additionally, our semi-conservative model can be interpreted in terms of cells infected by viruses, even though real viruses often have single stranded genomes that, on a molecular level, are conserved during replication. In particular, viral reproduction is *effectively* semi-conservative if the following assumptions apply:

1. Infected cells lyse (i.e. the “mother” cell dies) when viruses emerge from them.
2. The number of virus particles that emerge upon lysis is very large.
3. Of the large number of released virus particles, only a small number (*R_o_*) go on to infect other cells, independent of viral fitness. For simplicity, we assume that *R_o_ = 2* throughout this paper.

Assumptions 2 and 3 together insure that the same exact genome molecule that infects a cell does not initiate subsequent infections (i.e. that the process is essentially semi-conservative). Note that, when considering viruses, we keep track of the number of infected cells as opposed to free viruses, which cannot autonomously replicate. We assume that only a single genome infects a particular cell, i.e. low multiplicity of infection; without this assumption, a virus's fitness would depend not only on its own genotype, but also on that of co-infecting viruses [30]. Also note that our model does not explicitly consider infected versus uninfected cells [31]; for a treatment of lethal mutagenesis with such a model, see ref. [7].

### Flat, non-epistatic fitness landscape: survival phase versus extinction phase

The goal of this paper is to calculate which values of the population capacity (*N*) and mutation rate (*U*) support survival and which lead to extinction. In a sense, the answer is trivial: extinction is certain if *N<∞* and *U>0* since the population only has a finite number of configurations and all of them, including extinction, will be visited *eventually*. Nevertheless, the question remains as to which values of *N* and *U* enable populations to live a “long time” versus a “short time.” However, it is not clear *a priori* even whether there exists a sharp, qualitative distinction between “long” and “short” or whether those concepts continuously blur together. Obviously, a crucial prerequisite for understanding extinction in finite populations is to define exactly what is meant by “long” and “short,” i.e. “survival” vs. “extinction.” To this end, we first consider a simple, analytically solvable fitness landscape whose solutions clarify these crucial preliminary issues. Later, we consider a more realistic FL based on protein biophysics.

By “fitness landscape” (FL) we mean a mathematical function relating genotype to fitness. We first consider a very simple FL in which the distribution of fitness effects (DFE) among new mutations is always the same, independent of genotype and/or fitness; this FL is non-epistatic, by definition. To further simplify our analysis, we assume that this preliminary landscape is “flat,” insofar as all mutations are either lethal or completely neutral.

Given said assumptions, all relevant aspects of the population are completely described by the number of living cells (*n(t)*). *n(t)* thus undergoes a biased random walk, with a natural absorbing boundary at *n = 0* and a reflecting boundary at *n = N*. Since the number of lethal mutations per offspring is Poisson distributed with mean *Uλ*, the transition probabilities per unit time for increasing and decreasing *n* by a single individual are, respectively:
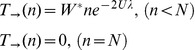
(1a)

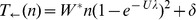
(1b)where W^*^ is the fitness of all viable cells and *δ* is the natural death rate. W^*^ plays no essential role, and is often set to one for convenience. Eqs.1 are plotted in fig.S1. The exponentials in eqs.1 express the probability that none or both of the offspring carry lethal mutations. Note the factors of *n* in eqs.1, which are not present in the simplest “text book” random walk. This model could be extended to handle bursts of *R* offspring by replacing factors of 2 with *R* and considering larger jumps.

We first investigate the average behavior eqs.1, and then perform a stochastic analysis. According to eqs.1 (see also Text S1), the expected change in the census *n* during the time interval *dt* (〈*d n〉*) obeys the following equation:
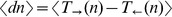
(2a)

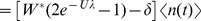
(2b)

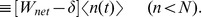
(2c)


**Figure 2:**
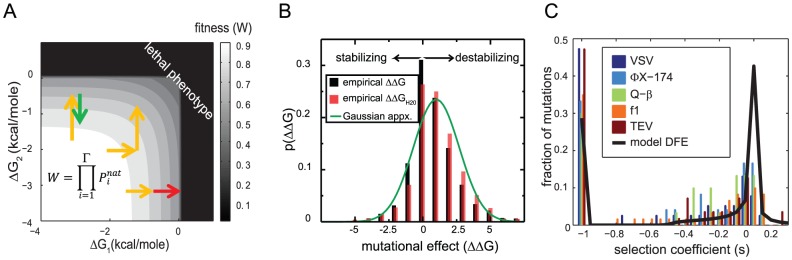
Biophysics-based fitness landscape (FL). **A:** A two dimensional slice of the *Γ* dimensional FL. Fitness values are shown in grayscale and pairs of mutations are represented by colored arrows. Deleterious mutations interact synergistically (yellow/red pair). Compensatory and non-epistatic mutations are also possible (yellow/green and yellow/yellow pairs, respectively). Mutations that push *ΔG>0* cause lethality. **B:** Distribution of mutational thermodynamic effects *p(ΔΔG)*. Our approximation for *p(ΔΔG)* agrees with experimental values obtained via thermal (black bars) and/or solute (red bars) denaturation. The ∼4,000 experimental values were taken from the ProTherm database [56]. **C:** The distribution of fitness effects (DFE) among new random mutations from our model (black curve) and several viral species (colored bars). The horizontal axis is the selection coefficient, which depends on fitness before and after the mutation: *s≡W_after_/W_before_−1*. The DFE from this model depends on *N* and *U* [27]; here *N = 10240, U = 2* (chosen so that the population was near the extinction threshold). See Methods and ref. [27] for procedures used to obtain the DFE.

We call *W_net_* the “net fitness,” since it has a component related to fitness of living cells (W*) discounted by a component that depends on the production rate (*Uλ)* of mutants. The most dramatic distinction between mean fitness and *W_net_* is that, while the former must be ≥0 (birth rates obviously can't be negative), the latter becomes negative when *Uλ>ln(2)* because of lethal mutations and semi-conservative replication. Note that for realistic values of *U* and *λ*, *W_net_* is substantially less than W^*^: e.g. if *U = 1* and *λ = 0.3* (see fig. 2c), W_net_ = 0.48W ^*^(i.e. mutation reduces fitness by 52%), which underscores the impact of lethal mutations and semi-conservative replication in limiting the growth of 〈*n*〉.

Eq.2 implies that the expected value (*〈n〉*) for the census size either grows (until *n = N*) or decays exponentially with rate *W_net_-δ*. We denote these two opposing regimes as the “survival phase” and “extinction phase.” The boundary between survival and extinction occurs when the natural death rate balances net fitness:
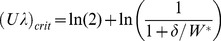
(3a)

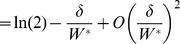
(3b)


Note that, if *Uλ>ln(2)≈0.7*, populations will be in the extinction phase even if no natural death occurs (*δ = 0*). To a close approximation, the effect of natural death is merely to decrease (*Uλ*)*_crit_* by an amount δ/W*, i.e. the number of natural deaths per generation. Also not that the value of *N* is irrelevant to whether populations are in the survival or extinction phase on this non-epistatic fitness landscape (FL); the picture will be radically different later, when we consider a more realistic FL.

We now turn to the stochastic features of this model. Given the initial condition that there are *N* cells at *t = 0*, all populations go extinct with probability one, but we can calculate the statistics of how long the population survives before going extinct (i.e. hitting the absorbing state at *n = 0*). In Text S1, we derive a general analytic formula for the mean time until extinction (*τ*), i.e. the mean “first passage time,” by approximating *n* as a continuous variable and solving differential equations. The continuity assumption is valid for *|v/D|≪1* (see Text S1). The asymptotic behavior of the general solution *τ(U,N)*, valid for large but finite *N* and *Uλ≠ln(2)*, is given by
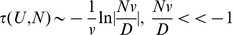
(4a)

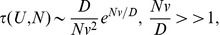
(4b)where 
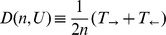
 and 
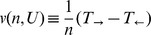
, plotted in fig.S1, can be interpreted as the diffusion coefficient and convection velocity from diffusion theory, respectively. Eqs.4, along with the exact analytic expressions, are plotted in fig.S2. Note that, since *v* equals the term in brackets in eq.2c, eqs.4a,b corresponds to the extinction and survival regimes, respectively.

The rough functional dependence of *τ* on *N* in eqs.4 might be anticipated intuitively. Eq.4a applies when populations are biased toward extinction. In that case one expects that *n(t)* decays exponentially from *n = N* down to *n = 0*: 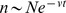 , which implies that 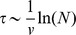, similar to eq.4A. On the other hand, eq.4b applies when populations are biased toward survival. In that case, one expects that extinction requires an extraordinary run of *∼N* lethal mutations, which should occur with probability on order of *(Uλ)^−N^*. The time until extinction in the survival regime thus might be expected to scale as 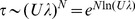 , similar to eq.4b which is also dominated by *N* in the exponent. In Text S1, we also analyze how *τ* scales with *U*. We find that in the survival phase, but not too far from the transition at *Uλ = ln(2)*, 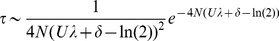. In the extinction phase we find 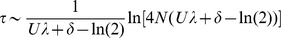. Thus, *τ* depends sharply on both *N* and *Uλ* in the survival phase, yet depends only weakly on these variables in the extinction phase. These approximations are plotted in fig.S2. In Text S1 and fig.S3, we also consider the variance in extinction time.

### Extinction on epistatic biophysical fitness landscape

We next consider a recently developed biophysics-based fitness landscape (FL), which features a continuous distribution of fitness effects (DFE) among random new mutations (fig. 2). The basic assumption of this approach is that mutations inflict a fitness penalty proportional to the extent that they unfold proteins by perturbing thermodynamic stability (*ΔG*). Below, and in fig. 2, we describe some important features of this model; see Methods and/or ref. [27] for details.

1. This FL is epistatic: a given mutation unfolds barely-stable proteins more so than very stable proteins.
2. Fitness increases (though usually very weakly) with increasing stability (i.e. decreasing *ΔG*).
3. Approximately 30% of all mutations are compensatory, although most increase fitness by only a negligibly small amount (*≪ 1/N*).
4. Each cell has a fixed number (*Γ*) of proteins, or, more precisely, *Γ* protein folding domains. For convenience, we assume *Γ = 20* in simulations.

Mutations that completely unfold proteins or hit a small fraction of functionally critical residues (e.g. the active site) are considered lethal. Points 1–3 above are not explicit assumptions of our model; rather, they follow naturally and implicitly from a biophysics-based framework (see Methods and ref. [27]). Crucially, the DFE from this FL roughly agrees with experimental data, at least for viruses [27] (fig. 2C). Unlike the flat landscape that we considered previously, the biophysical FL features many mutations that only slightly decrease fitness. These mutations profoundly increase the importance of *N* (even when *δ = 0*), because they can only be purged by sufficiently large populations (*|Ns|>1*).

**Figure 3:**
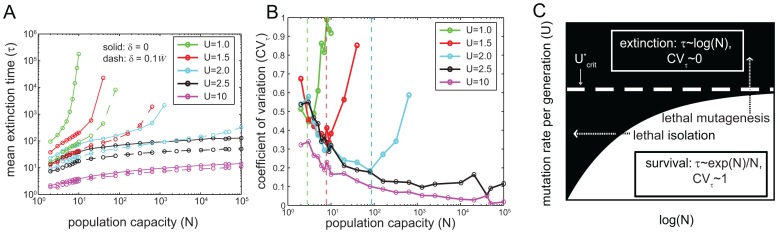
Extinction on biophysical fitness landscape. All finite populations eventually go extinct. The mean number of generations until extinction (*τ*) increases with population capacity (*N*) and decreases with mutation rate (*U*). **A**: *τ* versus *N* at various fixed *m*; notice the double logscale. Curves for *U<2.5* or so have an inflection point, signaling a qualitative transition from extinction to survival as *N* increases. Solid curves correspond to *δ = *0, while dashed curves correspond to δ/W* = 0.1. **B**: Coefficient of variation in time until extinction (*CV_τ_*) for the same parameters as panel A. *CV_τ_* increases towards one in the survival phase and decreases toward zero in the extinction phase, as *N* increases. Curves “peel off” toward *CV_τ_ = 1* at the critical population capacity (*N_crit_*), shown approximately with dashed lines. When 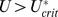, both simulation results and general arguments (see main text) show that curves do not peel off, i.e. *N_crit_* does not exist. **C**: Heuristic cartoon “phase diagram” summarizing the behavior from panels A,B. In panels A,B *τ* values are reported only in cases where extinction occurred within 10^5^ generations in each replicate. See main text and fig.S5 for a quantitative sense of how *N_crit_* depends on *U_crit_*. See Methods for averaging procedures. *Γ = 20* throughout this paper.

As with most multi-locus models, dynamics on our biophysics-based FL is too complex to solve analytically for finite *N*. Thus, we resort to stochastic computer simulations, as described in Methods. Fig. 3a shows how the mean extinction time (*τ*) depends on population capacity (*N*) for various mutation rates (*U*). On this log-log plot, upward bending curves increase faster than a power law (exponential-like scaling, c.f. eq.4b), whereas downward bending curves increase slower than a power law (logarithmic-like scaling, c.g. eq.4a). As we observed in the non-epistatic analytic model, *U* strongly impacts *τ*. Above an ultimate extinction rate (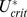
≈2.5, roughly estimated by eyeing simulation results), *τ* scales approximately logarithmically with *N* (extinction phase), even when *δ = 0* and *N→∞*. For very small *U*, *τ* scales approximately exponentially with *N* (survival phase) for all *N*. Fig.S4 explicitly shows *τ* versus *U*. For reference, we note that real RNA viruses have mutation rates in the approximate range *0.1<U<5*, whereas DNA based microbes generally have *U≈0.003* [32], [33]. According to fig. 3a, our model predicts that mutation rates characteristic of (non-mutator) DNA based microbes will always reside squarely in the survival regime for virtually any *N*, whereas RNA viruses lie near the extinction regime, and may be pushed into it, by modestly adjusting *N* and/or *U*. A few viral species have slightly higher mutation rates than the threshold 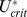 (≈2.5) from our model (e.g. bacteriophage Qβ has *U≈5* [33]); this is likely due to large burst size: see Discussion.

In stark contrast to the preliminary, non-epistatic FL, fig. 3a shows that *N*, not just *U*, also determines whether a population is in the survival or extinction phase. This is most apparent in the cyan curve representing *U = 2*, whose curvature suddenly changes near at a critical value (*N_crit_*). For large *N*, populations are in the survival phase, whereas below *N_crit_* populations enter the extinction phase. *N_crit_* becomes arbitrarily large as *U→U_crit_*. To get a quantitative sense of these values, given reasonable parameter values, consider *U = 2*: *N_crit_* equals *100* or so when *δ = 0* and rises to *∼10^4^* for the modest value of *δ = 0.1*. *τ* is only ∼100 generations in the extinction phase, and rises quickly from this level in the survival phase.

The extinction vs. survival phases are even more clearly delineated by the coefficient of variation (*CV_τ_*) of the extinction time (i.e. its standard deviation divided by its mean (*τ*)). *CV_τ_* measures stochasticity in populations' longevity. Fig. 3b shows that deep in the extinction phase, *CV_τ_→0*, whereas *CV_τ_→1* in the survival regime. These limits make intuitive sense: *CV_τ_ = 0* represents deterministic extinction, whereas *CV_τ_ = 1* is a hallmark of an exponential distribution describing the waiting time for an extraordinary run of independent lethal mutations in all *N* cells. Thus, *CV_τ_* behaves as an “order parameter” familiar from phase-transition theory: survival plays the role of the “ordered phase” (*CV_τ_ = 1*) while extinction represents the disordered phase (*CV_τ_ = 0*). In contrast, the preliminary, non-epistatic FL does not transition from the extinction to survival phase as *N* increases, since *CV_τ_* in a monotonic function of *N* in that model (fig.S3).

A crucial lesson from fig. 3b is that *N_crit_* depends on *U_crit_*, and vice versa. Curves representing higher mutation rates “peel off” to *CV_τ_ = 1* at larger values of *N* than do curves representing lower *U*. However, for 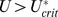, the curves cannot transition to *CV_τ_ = 1* for any value of *N*. This assertion is clear from the fact that a finite percentage (10% here, see Methods) of mutations are unconditionally lethal, and at sufficiently large *U*, nearly all progeny will acquire these (if no other) lethal mutations, resulting in extinction. Following the logic of eq.2, an upper bound for 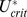 given 10% unconditional lethals, is 
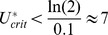
 nonsynonymous mutations per genome replication. Overall, the transition from extinction to survival is summarized by the “phase diagram” in fig. 3c. The non-rectangular phase boundary in fig. 3c emphasizes the interrelatedness of *N_crit_* and *U_crit_*. In particular, the boundary can be crossed by either increasing *U* (i.e. lethal mutagenesis) or decreasing *N*, which we refer to as “lethal isolation.” Fig.S5 shows a quantitative version of fig. 3c.

**Figure 4:**
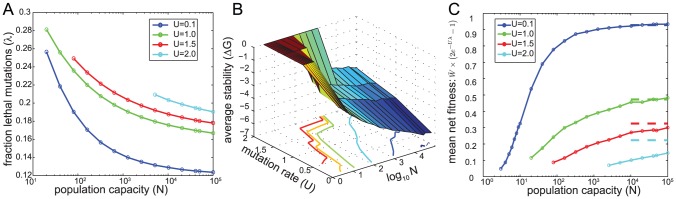
Small population capacity (*N*) and high mutation rate (*U*) cause depressed fitness, unstable proteins, and many lethal mutations. **A**: Fraction of mutations (*λ*) which were lethal during simulations on our biophysical fitness landscape. Fig.S6 shows a related plot of *Uλ/ln(2)* versus *N*. **B**: Protein stability (*ΔG*) averaged over both proteomes and populations. The accumulation of unstable proteins when *N* is small and/or *U* is large is the underlying cause of changes in *λ* observed in panel A. **C**: Mean net fitness, which takes into account both average birth rate and lethal mutations (eq.2c). Populations are not perfectly fit because of genetic drift (caused by small *N*) and mutation load (caused by large *U*). The classical expectation, which assumes *N = ∞* and no beneficial/compensatory mutations, predicts that overall growth rate is given by 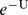 (shown with dashed lines on the right of panel B). The classical expectation fares poorly at small *N* and large *U*. Data is shown only for *(N,U)* values for which at least one of the replicate populations survived until the end of the simulation (see Methods); otherwise, *λ*, *W*, and *ΔG* are not clearly defined, since a quasi-steady state does not exist.

Why does the biophysics-based FL enable “lethal isolation” while the non-epistatic FL does not? The answer is that mean net fitness (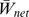) increases with *N* (fig. 4c) on the biophysics-based landscape; this increase derives from two distinct sources. First, larger populations more effectively purge weakly deleterious mutations having *Ns<1*, thereby driving up the mean birth rate (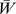) among living members of the population. Consequently, large populations can grow fast enough to outpace natural death (*δ*). Secondly, and more profoundly, small populations produce a larger fraction (*λ*) of lethal mutations (fig. 4a) on the epistatic, biophysics-based FL; these additional lethal mutations can decrease *W_net_* below zero and cause extinction even when *δ = 0* (figs. 3,4). The biophysical basis for this effect is that, when proteins are only barely stable (as predicted to be the case in small populations, see fig. 4b), more mutations are within striking range of the unfolding transition at *ΔG = 0* (fig. 2a) and a corresponding lethal phenotype.

## Discussion

A prerequisite for understanding extinction in finite populations is a coherent method for classifying extinction versus survival: Although all finite populations eventually go extinct, our analytic and simulation models show that “not all extinction is the same.” That observation led us to define two opposing dynamical phases for evolving populations: The extinction phase is characterized by rapid, nearly deterministic (*CV_τ_≈0*) decline whereas the survival phase is characterized by long yet uncertain extinction times (*CV_τ_≈1*). Intuitively, one usually thinks of small populations as being more stochastic than large populations. However, results from our biophysical fitness landscape (FL) show that that intuition needs refinement: the increased *stochasticity caused by small N actually makes extinction more deterministic* (e.g. *CV_τ_* can decrease with *N* in fig. 3b). The situation is analogous to a building experiencing an earthquake: if the strength of noisy seismic vibrations (i.e. genetic drift) crosses a threshold, gravity (i.e. deleterious mutations) deterministically destroys the building (i.e. population goes extinct).

Previous studies of mutation-induced extinction focused exclusively on either the role of high mutation rate (*U*) or small population capacity (*N*). Those that focused on high *U* neglected the role of genetic drift by assuming that *N = ∞* [2]–[4]. Likewise, those studies that included genetic drift generally neglected the role of high *U* [7], [19]–[21], [23], [34]. This paper bridges those previous two approaches by exploring how interplay between small *N* and large *U* accelerates extinction. We found that rapid extinction occurs on the biophysical FL whenever *N* is too small (*N<N_crit_*) or *U* is too large (*U>U_crit_*). Furthermore, we found that *N_crit_* depends on *U_crit_* and vice versa, i.e. the phase boundary in fig. 3c is not rectangular. In particular, small population capacity reduces *U_crit_*. This reduction is generally modest: e.g. we see *U_crit_* shift from about two to about one in figs. 3 and S5, as *N* varies from 10 to 10^5^. Although *U_crit_* depends only weakly on *N*, fig. 3 shows that *τ*, as well as the qualitative behavior of the population, can depend dramatically on *N*.

Semi-conservative reproduction is a key feature of our model that distinguishes it from most previous studies, e.g. refs. [2], [19], [20], [22], [23]. This distinction is sharpest in the regime that *δ = 0*, where low fitness (i.e. long generation time) contributes to extinction only insofar as it increases the fraction of lethal mutations (*λ*). In other words, the population is its own (and only) enemy in the *δ = 0* regime. In reality, populations must survive not only in spite of themselves, but also in spite of death imposed by the environment. Fig. 3a shows that, in the plausible scenario where 10% of the population dies from natural causes each generation, *τ* is further shortened significantly. We note that obtaining parameter values for *δ* is not always straightforward because it requires discriminating between natural death and density-dependent death (i.e. death due to fixed *N*). For example, cells infected with HIV turn over approximately once per day [35], but it is unclear what fraction of the turnover is due to density dependence versus other “natural” causes.

For the sake of simplicity, we assumed that only two offspring result from a birth event. In the case of viruses, two should be replaced by an “effective burst size” that takes into account the number of virions released during the infected cell's lifetime as well as the fraction of those virions that go on to infect future cells. A simple deterministic analysis [2] suggests that *U_crit_* increases as the logarithm of effective burst size, but otherwise does not change the qualitative picture. In the context of finite populations, large burst size may also non-trivially reduce the effective population size [36], since all members of a burst are closely related; we leave investigation of this topic to future work.

Apart from large burst size, Martin and Gandon [7] recently pointed out another mechanism that may partially buffer viral populations against extinction. Using an explicit viral dynamics model that includes both susceptible and infected cells, those authors point out that as viral load declines under elevated mutation rates, the number of susceptible cells is predicted to correspondingly increase. This effect may tend to offset and/or halt the decline in growth rate caused by elevated mutation rates. While this mechanism may be important, our model predicts that its extinction-buffering potential may be limited. In particular, we predict that the fraction of lethal mutations (*λ*) increases as fitness decreases (fig. 4a); in our model, any increased growth rate, from whatever origin, will be countered by a correspondingly elevated death rate from lethal mutations.

### Previous calculations of *U_crit_* (deterministic studies)

Deterministic models remove *N* from consideration by assuming that *N = ∞*, which enables a comparatively straightforward calculation of the ultimate mutation rate (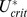) beyond which even infinitely large populations go extinct. Our biophysical model also features an ultimate mutation rate (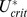) (see horizontal asymptote in fig. 3c), and additionally it predicts that when *N* is finite, 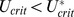. While it is unsurprising that *τ* decreases as *N* does, it *is* rather surprising that decreasing *N* can fundamentally change the dynamical regime of the population from survival to extinction.

Zeldovich et al. [3] utilized a biophysical fitness landscape similar to the one presented here. Apart from their assumption that *N = ∞*, the main difference with our approach is that their fitness landscape had a strictly flat “mesa,” i.e. they approximated eq.6 (Methods) as a true step function. By contrast, our model features nearly-neutral mutations (fig.S4) which enhance the role of population capacity (*N*), since mutations with *Ns<1* are invisible to natural selection [27], [37].

The deterministic theory of Bull and Wilke, first laid out in ref. [2] and subsequently elaborated upon in ref. [4], is another important benchmark for comparison. Using a simple, classical equation, those authors calculated equilibrium mean fitness and compared this to the rate of natural death. Neglecting beneficial/compensatory mutations, they calculated a maximum allowable “deleterious mutation rate” of *ln(2)≈0.7*, which is the same value we calculated for the *lethal* mutation rate (*Uλ*) in the preliminary, non-epistatic FL. By contrast, on our biophysical FL, we predict an *overall* maximum nonsynonymous mutation rate of 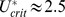 (fig. 3). The discrepancy between 0.7 and 2.5 derives from many factors, including compensatory mutations in our model and ambiguity in what those authors mean by “deleterious,” i.e. which mutations they would define as deleterious as opposed to neutral.

### Previous calculations of *N_crit_* (stochastic studies)

Random drift is the paramount concern of a separate line of previous studies that describe extinction in terms of Muller's ratchet [19]–[22], [38]. However, those studies minimize the importance of mutation rate. For example, neglecting beneficial mutations and using an approach based on fixation probabilities, Lande [21] calculated that *τ∼1/U*; i.e. his result is that *U* merely sets the time units but is irrelevant to the essential behavior. As another example, Whitlock [23] included beneficial mutations and calculated that *N_crit_∼(U_deleterious_/U_beneficial_)^1/3^*, which depends only on the balance of beneficial to deleterious mutations and *not* on the mutation rate itself. Both of those examples contradict our results, which show that *N_crit_* and *τ* depend dramatically on *|U|*. The dominant reason for the discrepancy is that those authors assumed that deleterious mutations occur “one at a time,” which is not true when the rate that mutations are introduced (*U*) exceeds the rate at which selection removes them *(∼1/s*). When *U/s≫1*, the population experiences “Hill-Robertson interference” [39], which both accelerates extinction and also makes analytic solutions intractable.

A separate, very serious concern about many previous studies (e.g. refs. [19], [21]) is that, for all parameter values they explored, they always observed a small coefficient of variation in extinction time (*CV_τ_*). Based on our results (fig. 3), this suggests that *those authors only probed the extinction regime*. In other words, their models were constructed such that extinction occurred nearly deterministically. By contrast, it seems likely that most, if not all, natural populations are in the survival regime as long as their population size and/or mutation rate are not interfered with externally (e.g. via mutagens or habitat destruction). A related issue concerns the initial conditions of those models. They assumed that populations were extremely fit initially, such that each individual leaves a large number (*R_o_*) of descendants (*R_o_≫1*). Extinction occurs in those models when *R_o_* semi-deterministically drops to just below one, after several deleterious mutations achieve fixation. By contrast, our simulations begin in a natural condition (see Methods): mutation-selection-drift equilibrium, which may not even exist in those previous models. The existence of a quasi-equilibrium state, i.e. the survival phase, is a major advantage of our approach. Indeed, the survival phase can be viewed as a stochastic analog of deterministic mutation-selection equilibrium.

### Other sources of random genetic drift

In this paper we have focused on the subtly deleterious impact of unbiased fluctuations on allele frequency (i.e. genetic drift) caused by finite population capacity (*N*). In addition to finite *N*, several other factors can have a similar effect, including population bottlenecks, micro-environmental fluctuations, and stochasticity in gene expression [40]; these effects are sometimes summarized collectively by an “effective population size” (*N_e_*) [36]. Of particular relevance to extinction of RNA viruses are population bottlenecks that occur during transmission events. Indeed, it is estimated that most HIV-1 infections originate from a single infectious particle, which would greatly reduce *N_e_* below the viral load.

### Importance of fitness landscape

An important result from previous studies is that *τ* depends strongly on both the severity of deleterious mutations (i.e. the DFE [19], [21], [34]) and on epistasis [22], [38], [41]. The DFE has traditionally been represented by either a single selection coefficient (i.e. a Dirac delta function) or by a continuous function (e.g. Gamma distributions), which was assumed not to change with fitness and/or time. Thus, even relatively simple approaches were forced to make somewhat ad-hoc modeling choices and also introduce several parameters. The fact that *τ* depends strongly on the DFE thus presents a dilemma to researchers: they must either comb through a high-dimensional parameter space or else their results depend on myriad questionable assumptions. Our model circumvents this problem because the parameters (e.g. eqs. 6,7 in Methods) are not “adjustable”; rather, they are set by strictly biophysical considerations. In effect, we exchanged a poorly understood, high-level question (how mutations affect fitness) for a well-understood, microscopic question (how mutations affect protein folding thermodynamics). The validity of this exchange is commensurate with the extent to which our DFE matches experimental data (ref. [27] and fig.S4).

Apart from the advantages of our FL, we expect that the qualitative behavior in fig. 3 might also be observed in some traditional models. We anticipate that the essential requirements are (i) both beneficial and deleterious mutations (so that the population does not inevitably “slide downhill”) and some upper bound on fitness (so that the population cannot forever “climb uphill”).

### Extinction versus “error catastrophe”

Decades ago, Eigen calculated that genomes can become “delocalized” in sequence space during an “error catastrophe” when the mutation rate exceeds a critical value [42]. As pointed out by previous authors [2], extinction and error catastrophe are distinct concepts: The former is a demographic process whereas the latter refers to loss of the single fittest genotype on a toy fitness landscape, usually in the *N = ∞* limit. Nevertheless, some results from one study of error catastrophe in the context of finite *N* [43] hint at our results in fig. 3; e.g. they observed that the time taken for populations to experience delocalization decreases with *N*.

### Connections with experiments and viral infection treatment

In reality, is extinction accompanied by excessive lethal mutations (as in our model) or merely by a slow generation time that is unable to keep pace with natural death (as in previous models, e.g. refs. [2], [19], [20], [22], [23]? These two scenarios are distinguishable in laboratory evolution experiments on viruses because viral load (nucleic acid molecules per mL) and infectivity (plaques formed per mL viral suspension) can be measured separately. Several experiments on at least three viral species [29], [44]–[47] show that when *U* is elevated near/past *U_crit_*, viral load transiently continues to increase, simultaneous with a decline in infectivity. Thus, noninfectious genomes (i.e. those carrying lethal mutations) signal extinction during experiments, in accord with our model's interpretation of extinction.

A clinically relevant prediction of our model is that changing *N* can radically alter population survival, especially when *U* is elevated by drugs. This phenomenon was observed experimentally [8] with foot-and-mouth disease virus, where merely 10-fold dilutions during viral passages dramatically accelerated extinction in the presence of mutagenic drugs. As remarked by those authors, this finding suggests that therapies combining both mutagenic drugs and traditional drugs (which reduce the number of viable viruses) could substantially increase efficacy. Indeed, our analytic results (eqs.4 and eq.S11a) imply that even in the survival phase, the expected time to extinction depends exponentially on (i.e. is very sensitive to) both *U* and *N*, suggesting that altering either of these parameters could dramatically impact the chances of population extinction during a fixed time interval.

Apart from extinction per se, our general biophysics-based approach also has substantial experimental support. Our basic assumption is that protein unfolding/misfolding accounts for the deleterious effects of most mutations. If this were true, species with high *U* and/or low *N* should have less stable proteins. Several experimental facts suggest that this is in fact the case. First, chaperone overexpression compensated for the fitness decline caused by single-cell bottlenecks (low *N*) in bacterial populations [48], [49]. Thus, these populations likely contained unstable, unfolded proteins which caused the fitness decline. Secondly, Fernandez and Lynch [50] recently reported more structural defects and thermodynamic instability among monomeric protein subunits in small populations than in large populations. Along similar lines, another study calculated less stability among proteins in endosymbiotic bacteria (small *N*) than in orthologs from free living relatives (large *N*) [51]. Thirdly, proteins from RNA viruses (high *U*) have a lower density of van der Waals contacts than orthologs in DNA viruses (lower *U*), suggesting, though not proving that RNA viral proteins are less stable [52]. Indeed, we have gone even further and predicted the distribution of stabilities within proteomes from species with various *U* and *N* (see fig. 5 from ref. [27]).

## Methods

### Biophysics-based fitness landscape

The approach here closely follows ref. [27]. Every cell contains a number (*Γ*) of *well-adapted* proteins, each of which exists in thermal equilibrium between its native, functional conformation and an ensemble of unfolded, nonfunctional conformations. The fraction of time in equilibrium that protein *i* spends in its native conformation is 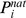. Qualitatively, our assumption is that fitness is impaired when either the concentration of folded proteins decreases or, equivalently, the concentration of unfolded proteins increases. Quantitatively, we assume that
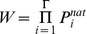
(5a)

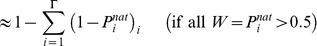
(5b)

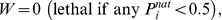
(5c)where 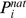 is the fraction of time in equilibrium that protein *i* spends in its native conformation and *W* is fitness (i.e. birth rate). The approximation between eq.5a and eq.5b is valid for *P^nat^≈1*, as is the case for real proteins (see below). Eq.5a emphasizes the positive interpretation that each of the proteins is required in order for the organism to live and function. Likewise, eq.5b emphasizes the negative interpretation that misfolded/unfolded protein hurts the organism in proportion to their concentration in the cell.

In our model, *P^nat^* is the master variable that connects proteins with fitness. However, it is simpler to work with a closely related quantity: the free energy difference (*ΔG*, also called “protein stability”) between the folded conformation and the ensemble of nonfunctional conformations. We assume that proteins fold “two-state” [53], which implies the relationship
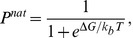
(6)where *k_b_* is Boltzman's constant and *T* is temperature.

Working with *ΔG* simplifies mutational effects because, (i) effects of mutations on free energy (*ΔΔG*) are well characterized experimentally and (ii) *ΔΔG* (but not *ΔP^nat^*) is additive when several mutations accumulate sequentially [54], [55]. We approximate *p(ΔΔG)* by a Gaussian function with a mean of +1 kcal/mole and standard deviation 1.7 kcal/mole [3], [56], which reasonably matches empirical data from the ProTherm database [56] (fig.S4):
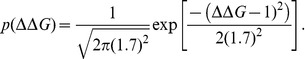
(7)


We also assume that *p(ΔΔG)* is independent of *ΔG*, which is consistent with empirical data [5], [56], though only to a rough approximation.

While most mutations in our model only alter *ΔG*, a small fraction destroy protein function for non-thermodynamic reasons. For example, a few amino acid positions comprise the active catalytic site, and virtually all mutations there will abolish functional activity. Assuming that there are 3 catalytic residues, another 3 nearby critical sites and 100 total residues in the folding domain, these represent 6% of all random mutations. Besides the active site, some point mutations abolish activity by introducing premature STOP codons. Assuming random codon usage, premature STOP codons represent ≈4% of random mutations [27]. Thus, together, these categories comprise ≈10% of all nonsynonymous mutations, which we assume to unconditionally confer a lethal phenotype.

Eqs.6,7 al ong with said assumptions regarding lethal mutations, indirectly imply the distribution of fitness effects (DFE) in the biophysical model; detailed explanation of how this works is the subject of ref. [27]. Briefly, we first equilibrated populations for at least 10^5^ generations, at which point populations had substantial diversity in fitness. Next, we measured the DFE among all single point mutations for each clone in the population. Finally, these DFE were averaged to obtain the overall DFE, e.g. in fig. 2C. This procedure essentially averages the DFE of each clone, weighting each in proportion to its probability of being randomly chosen as the starting point for mutagenesis experiments. Since our DFE describes nonsynonymous mutations only, synonymous mutations were removed from the experimental datasets in fig. 2c.

### Simulation procedures

We iterated the birth-death-mutation process for 10^5^ generations or until population extinction, whichever occurred first. Each birth event represents *1/n(t)* generations. All populations were initialized with genomes (i.e. sets of *ΔG* values) sampled from a single, “burn-in” population that had previously achieved mutation-selection-drift equilibrium during 10^5^ generations of evolution. The parameter values (*N = 10^5^, U = 1*) of the burn-in population were chosen so as to lie clearly in the survival regime yet close to the regions of parameter space being probed throughout the paper. This choice minimizes the impact of (inherently somewhat artificial) initial conditions. The fraction of lethal mutations (*λ*) was estimated during each simulation run as the total number of lethal mutations divided by 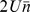, where 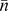 is the time-averaged number of cells during the run.

## Supporting Information

Figure S1 Transition probabilities, “convection velocity” and “diffusion coefficient” as functions of the lethal mutation rate (*Uλ*), assuming that *δ = 0*. These are the per capita quantities (i.e. *n = 1*).
(EPS)

Figure S2 Mean time until extinction on flat, non-epistatic fitness landscape. Solid curves illustrate the exact solution, eq.S11. The dashed curves in panel A illustrate the approximations eqs.S12A,S12B. Dashed curves in panel B illustrate eq.S13A. The vertical dotted line marks the transition at *Uλ = ln(2)*. *δ = 0*. Note that eq.S13A breaks down for very small *Uλ*. As discussed in the text, the entire continuum approach breaks down in that regime.
(EPS)

Figure S3 Coefficient of variation (standard deviation divided by mean) in time until extinction (*CVτ*) when *δ = 0*. A: Supercritical (*Uλ>ln(2)*) populations become more deterministic as *N* increases, while subcritical populations become more stochastic. B: *CVτ* decreases sharply at *Uλ = ln(2)*, though the sharpness of the transition increases with *N*.
(EPS)

Figure S4 The mean time until extinction declines rapidly as *U* increases when *U<Ucrit*. Data here is a subset of that shown in fig. 3a from the main text.
(EPS)

Figure S5 Quantitative version of fig. 3c from the main text. As in fig. 3c, here we see a boundary between phases that increases up and to the right. However, fluctuations at very low *N* inevitably obscure the underlying phase boundary. Points in the survival regime were colored white (i.e. we assumed *CVτ = 1*) if extinction never occurred during simulations during a feasible amount of time (10^5^ generations). *δ = 0*.
(EPS)

Figure S6 Lethal mutations during simulations on our biophysical landscape when *δ = 0*. As in fig. 4 from the main text, this plot shows data only from (*N,U*) pairs such that populations survived for the duration of simulations (105 generations). However, the left-most terminus of each curve is near the extinction phase. In our non-epistatic, analytical model, the boundary between survival and extinction phases occurs at *Uλ = ln(2)*. Here we see that the criterion that *Uλ = ln(2)* is unlikely to mark the transition to extinction on our (epistatic) biophysical model, since the left-most termini always have *Uλ* substantially below *ln(2)*.
(EPS)

Text S1 Analytical derivations of mean and variance in extinction time on flat, non-epistatic fitness landscape.
(PDF)
