# Supplementary material for: Mutation Induced Extinction in Finite Populations: Lethal Mutagenesis and Lethal Isolation
Source: PLoS Comput Biol. 2012 Aug 2;8(8):e1002609. doi: 10.1371/journal.pcbi.1002609 (PMC3410861; doi:10.1371/journal.pcbi.1002609)
Supplement: Text S1 — Analytical derivations of mean and variance in extinction time on flat, non-epistatic fitness landscape. (PDF) [file pcbi.1002609.s007.pdf]

Mutation induced extinction in finite populations: lethal mutagenesis and lethal isolation

Supplementary text

Dynamics of average census ($\langle n(t) \rangle$) on flat, non-epistatic landscape

In our analytic approximations, we assume that all (living) members of the population (i) are equally fit and (ii) have the same fraction (λ) of lethal mutations. In that case there are no genotypes to speak of, and the population is completely described by its census size (*n(t)*). During a small time *dt*, the population size increases if a birth occurs and neither offspring carry a lethal mutation. Likewise, if both offspring carry one or more lethal mutations, or if a natural death occurs, the population size decreases by one. These processes are represented by the transition probabilities (same as eqs. 1, main text):

$$T_{\rightarrow}(n) = n\, W^*\, (e^{-U\lambda})^2 = n\, W^*\, e^{-2U\lambda} \qquad n < N \qquad \text{[S1A]}$$

$$T_{\rightarrow}(n) = 0 \qquad n = N$$

$$T_{\leftarrow}(n) = nW^*\, (1 - e^{-U\lambda})^2 + n\delta, \qquad \text{[S1B]}$$

where $U\lambda$ is the expected number of new lethal mutations per genome and W^*, henceforth set to one, merely sets the generation time. Note that $T_{\rightarrow}$ and $T_{\leftarrow}$ depend on *n* because the total number of births during *dt* is proportional to the current population size. Eqs. S1A,S1B are plotted in fig. S1.

The expected change in census (*dn*) at time *t*, given that the census equals n_o, is given by

$$E[dn|n_o] = +1\ \cdot T_{\rightarrow}(n_o)dt - 1 \cdot T_{\leftarrow}(n_o)dt. \qquad \text{[S2]}$$

Taking the expectation of both sides with respect to n_o and plugging in eqs.1 for the transition probabilities, we obtain the unconditional expectation of *dn*, yielding

$$\langle dn \rangle = \left(2\, e^{-U\lambda} - 1 - \delta\right)\langle n \rangle \equiv (W_{net} - \delta)\langle \text{ n} \rangle, \qquad \text{[S3]}$$

whose solution is obviously $\langle n \rangle \sim exp[(W_{net}\text{-}\delta)t]$. Note, however, that the actual trajectory of *n(t)* often does not resemble the average trajectory $\langle n(t) \rangle$, e.g. when extinction occurs.

Mean time until extinction (τ) on flat, non-epistatic fitness landscape

We want to calculate the mean time (τ) until extinction, given that the population has n individuals at $t=0$ and cannot ever exceed a fixed size N. Following Van Kampen [1] (XII.2, XII.3) we begin by writing a recursion relation for the probability density ($f_n(t)$) that the population goes extinct at time t, given that it initially has size n:

$$f_n(t) = dt\, T_{\rightarrow}(n) f_{n+1}(t-dt)$$

$$+dt\, T_{\leftarrow}(n) f_{n-1}(t-dt)$$

$$+(1-dt\, T_{\rightarrow}(n) - dt\, T_{\leftarrow}(n)) f_n(t-dt) \qquad [S4]$$

Eq.S4, valid for $2<n<N$, merely states that if the population is to go extinct after t generations, it must first either increase its size by one, decrease its size by one, or else remain at size n. This difference equation can be cast as a more tractable partial differential equation by Taylor expanding to second order in n and first order in t, which yields

$$\frac{\partial f}{\partial t} = \left(\frac{T_{\rightarrow} + T_{\leftarrow}}{2}\right)\frac{\partial^2 f}{\partial n^2} + (T_{\rightarrow} - T_{\leftarrow})\frac{\partial f}{\partial n}. \qquad [S5]$$

Later we will consider when the continuum approximation is valid and when it breaks down. Since we're interested mainly in the mean extinction time (and not the entire distribution), our task is further simplified by multiplying eq.S5 by t and then integrating over t. Integrating by parts on the LHS, we obtain

$$-1 = Dn\frac{d^2\tau}{dn^2} + vn\frac{\partial \tau}{\partial n}, \qquad [S6]$$

where

$$D(U) \equiv \frac{1}{n}\left(\frac{T_{\rightarrow} + T_{\leftarrow}}{2}\right)$$

$$v(U) \equiv \frac{1}{n}(T_{\rightarrow} - T_{\leftarrow})$$

are independent of n and analogous to the diffusion coefficient and convection velocity familiar from diffusion theory. Fig.S1B illustrates how D and v depend on U.

Eq.S6 is merely a linear ordinary differential equation, which is easily solved by first multiplying by the "integrating factor" $e^{nv/D}$. The boundary conditions are $\tau(n=0)=0$ (i.e. a population that begins with zero individuals immediately goes extinct) and $\left.\frac{d\tau}{dn}\right]_{n=N} = 0$, which symbolizes the "reflecting boundary" at $n=N$ [1]. The resulting formal solution is

$$\tau(n) = \frac{1}{D}\int_0^n dn' e^{-n'v/D}\int_{n'}^N dn'' \frac{e^{n''v/D}}{n''}. \qquad [S7]$$

Eq.S7 can be simplified by introducing the non-dimensional variables $x \equiv nv/D$, $x_{max} \equiv Nv/D$. Then,

$$\tau(x, x_{max}) = \frac{1}{v} I(x, x_{max}), \qquad \text{[S8]}$$

where $I(x, x_{max})$ is the dimensionless integral

$$I(x, x_{max}) \equiv \int_0^x dx' \, e^{-x'} \int_{x'}^{x_{max}} dx'' \frac{e^{x''}}{x''}$$

$$= \int_0^x dx'' \frac{e^{x''}}{x''} \int_0^{x''} dx' \, e^{-x'} + \int_x^{x_{max}} dx'' \frac{e^{x''}}{x''} \int_0^x dx' \, e^{-x'}$$

$$= \int_0^x dx'' \frac{e^{x''}}{x''} (1 - e^{-x''}) + (1 - e^{-x}) \int_x^{x_{max}} dx'' \frac{e^{x''}}{x''}$$

$$= \int_0^x dx'' \frac{e^{x''} - 1}{x''} + (1 - e^{-x}) \int_x^{x_{max}} dx'' \frac{e^{x''}}{x''}, \qquad \text{[S9]}$$

The primes in eq.S9 merely represent dummy variables of integration. Eq.S9 can be further simplified by introducing the "exponential integral function" $E_i(x) \equiv \int_{-\infty}^{x} dy \frac{e^y}{y}$. Then,

$$I(x, x_{max}) = E_i(x) - E_i(0) - \ln|x| + \ln 0 + (1 - e^{-x})[E_i(x_{max}) - E_i(x)].$$

Neither $E_i(0)$ nor $\ln(0)$ exist, but these two terms cancel near zero since $\lim_{x \to 0} E_i(x) = \ln|x| + \gamma$ [2], where $\gamma \approx 0.577$ is the Euler-Mascheroni constant [2]. Thus,

$$I(x, x_{max}) = E_i(x_{max}) - e^{-x}[E_i(x_{max}) - E_i(x)] - \ln|x| - \gamma \qquad \text{[S10]}$$

Of particular interest is the case $x=x_{max}$, which provides the mean extinction time in the case that the population is initially full (i.e. $n=N$ at $t=0$):

$$\tau(N) = \frac{1}{v}\left[E_i\left(\frac{Nv}{D}\right) - \ln\left|\frac{Nv}{D}\right| - \gamma\right]. \qquad \text{[S11]}$$

Eq. S11 is more illuminating in its limiting forms. In particular, for large values of its argument, $E_i(x) = \frac{e^x}{x} + O(1/x^2)$, while for small x, $E_i(x) = \ln|x| + \gamma + x + O(x^2)$. Thus,

$$\tau(N, U) \sim \frac{D}{Nv^2} e^{\frac{Nv}{D}} \qquad \frac{Nv}{D} \gg 1 \qquad \text{[S12a]}$$

$$\tau(N, U) \sim -\frac{1}{v} \ln\left|\frac{Nv}{D}\right| \qquad \frac{Nv}{D} \ll -1 \qquad \text{[S12b]}$$

$$\tau(N, U) \sim \frac{N}{D} \qquad \left|\frac{Nv}{D}\right| \ll 1 \qquad \text{[S12c]}$$

Eqs. S12a, S12b are the same as eqs. 3a,3b from the main text. τ scales nearly exponentially with N in the survival regime (eq.S12a) and logarithmically with N in the extinction regime (eq.S12b). In the marginal case where $v=0$ (i.e. $m= \ln(2)$), $\tau \sim N$, which can be contrasted the familiar result that time scales as distance squared (i.e. N^2) for simple diffusion. The reason that $\tau \sim N^1$ here is that $T_{\rightarrow}$ and $T_{\leftarrow}$ are proportional to n in the present case. Also note that D/v, which depends only on $U\lambda$, sets a characteristic scale for the population size. For "small" populations, $N \ll D/v$ and $\tau \sim N$, according to eq.S12c. As $v \rightarrow 0$ (i.e. $U \rightarrow \ln(2)$), D/v diverges and all populations behave as if they were "small." For finite v, $|Nv/D|$ is usually much greater than

one for realistic population sizes, and eqs. S12a, S12b are valid approximations. Fig.S2 illustrates eq.S11, along with the approximations (eqs.S12).

Eqs.S12 specify how τ scales with *N*, but we would also like to know how τ scales with $U\lambda$. To do this, we expand *v/D* and *v*, which are both smooth functions (see fig.S1B), in powers of $U\lambda$-*ln(2)*. Then,

$$\tau(N,m) \sim \frac{1}{4N(U\lambda+\delta-\ln(2))^2} e^{-4N(U\lambda+\delta-\ln(2))} \qquad U\lambda+\delta<\ln(2) \qquad \text{[S13a]}$$

$$\tau \sim \frac{1}{U\lambda+\delta-\ln(2)} \ln[4N(U\lambda+\delta-\ln(2)] \qquad U\lambda+\delta>\ln(2) \qquad \text{[S13b]}$$

Validity of continuum approach

As shown in fig.S3B, eq.S13A provides an excellent approximation as long as $U\lambda$ is not too small. However, there is a much more serious problem in the small $U\lambda$ regime: the *entire* continuum approach breaks down. In some sense the breakdown is obvious; in reality τ must approach infinity as $U\lambda \rightarrow 0$, yet the exact solution (eq.S11) to the continuum approach yields a (large) finite value in this limit. The continuum approximation that bridges eqs.S4,S5 is valid when the distribution of extinction times ($f_n(t)$) obeys

$$f_{n\pm 1}(t) \approx f_n(t) \pm f'_n(t) + \frac{1}{2} f''_n(t) \qquad \text{[S14]}$$

We can check the self-consistency of the continuum approach by plugging in an approximate solution *f(n,t)* to eq.S5 and noticing when eq.S14 breaks down. Since all populations eventually go extinct, the steady state solution $f(n,\infty)$ equals one, for all *n*. However, for $m \approx 0$, the decay time to this solution is long, and we expect that $f(n,\infty) \sim e^{-nv/D}$. Indeed, this approximation satisfies eq.S5, though it does not *quite* satisfy the appropriate boundary condition that $f'(N,\infty) = 0$, since $e^{-Nv/D}$ is not quite zero. Plugging this exponential form into eq.S14, we see that eq.S14 requires that $v/D \ll 1$, which is true near the lethal mutagenesis transition, but not deep in the survival regime. Since survival times are astronomically large deep in the survival regime, we expect the continuum approximation to accurately describe situations of biological interest (where extinction may occur on human timescales).

Coefficient of variation in extinction time (CV_τ)

Returning the the continuum framework, we can also derive an equation for the mean square time until extinction ($\langle t^2 \rangle \equiv Q(N)$) by multiplying both sides of eq.S5 by t^2, then integrating by parts, which yields

$$-2\tau(n) = DnQ'' + vnQ'. \qquad \text{[S15]}$$

Defining $x \equiv nv/D$, as before, the formal solution for *Q(N)* is

$$Q(N) = \frac{2}{v}\int_0^{x_{max}} dx' e^{-x'} \int_{x'}^{x_{max}} dx'' \frac{e^{x''}}{x''}\tau(x'')$$
$$= \frac{2}{v}\int_0^{x_{max}} dx'' \frac{e^{x''}}{x''}\tau(x'') \int_0^{x''} dx' e^{-x'}$$
$$= \frac{2}{v}\int_0^{x_{max}} dx'' \tfrac{1}{x''}\left(e^{x''} - 1\right)\tau(x''), \quad [S16]$$

where $\tau(x)$ is given by eq.S10 (not eq.S11) and recalling that $\tau(x) \equiv I(x)/v$. It is helpful to partition τ into two terms: one that depends only on x_{max} ($\tau(x_{max})$, given by eq.S11), and a remainder $R(x)$.

$$R(x) \equiv -e^{-x}[E_i(x_{max}) - E_i(x)] - \ln(x/x_{max}) \quad [S17]$$

Then,

$$Q(N) = 2\tau^2(x_{max}) + \tfrac{2}{v^2}\int_0^{x_{max}} dx \frac{1}{x}(e^x - 1)R(x).$$

Therefore,

$$CV_\tau(N) = \left[1 + \frac{2}{v^2\tau^2(x_{max})}\int_0^{x_{max}} dx \frac{1}{x}(e^x - 1)R(x)\right]^{1/2}. \quad [S18]$$

The integrals in eq.S18 are not straightforward, but they can be evaluated numerically. Fig.S3 illustrates how $CV\tau$ depends on N and $U\lambda$. Below the lethal mutagenesis threshold ($U\lambda < ln(2)$), populations become more stochastic as N increases, since $CV\tau \rightarrow 1$. $CV\tau=1$ corresponds to an exponential process of waiting for a run of extraordinary bad luck, where all N cells independently acquire lethal mutations. In contrast, above the threshold, populations become less stochastic as N increases, since $CV\tau$ decreases. This corresponds to the fact that large populations deterministically go extinct at $U\lambda > ln(2)$. At exactly the critical mutation rate ($U\lambda = ln(2)$), $CV\tau$ can be evaluated analytically, using eqs.S12C,S18: $CV\tau = \sqrt{(2)}/2$ for all N.

References

1. Kampen NGv (1992) Stochastic processes in physics and chemistry. Amsterdam ; New York: North-Holland. xiv, 465 p. p.

2. Bender CM, Orszag SA (1999) Advanced mathematical methods for scientists and engineers. New York: Springer. xiv, 593 p. p.
